# The feeling of “*Urami*”: A structural topic modeling approach

**DOI:** 10.1371/journal.pone.0349193

**Published:** 2026-05-26

**Authors:** Hikaru Koike, Hirohito Okano, Michio Nomura

**Affiliations:** Graduate School of Education, Kyoto University, Kyoto, Japan; South Australian Health and Medical Research Institute Limited, AUSTRALIA

## Abstract

“*Urami*” is the negative emotional state that one experiences when treated unfairly by others. While similar to anger, it differs in various ways, such as in its long-term persistence. Empirical studies on *urami* have suggested that it comprises factors such as feelings of injustice, unforgiveness, and helplessness. However, the generalizability of its factor structure is questionable because most previous studies were based on small sample sizes. In this study, free descriptive responses regarding *urami* and anger provided by 489 participants (aged 19–72 years) were quantified and analyzed using structural topic modeling, a type of natural language processing algorithm. The results revealed the topics that compose the conceptual structure and the situations in which *urami* occurs, as well as the relationships between some grouped situations and individual traits, such as narcissism. Furthermore, comparing the conceptual structure and situational occurrences of *urami* and anger based on quantitative criteria revealed unique elements of *urami,* such as emotional persistence. These elements are particularly likely to occur when one is victimized by a close other.

## Introduction

Intense negative emotions directed toward others can cause aggressive behavior and adversely affect one’s own mind and body. The word “*urami*” has been used to express this emotional state since ancient times in Japan. In everyday circumstances, *urami* may be experienced when another person’s words or actions are perceived as having led to circumstances that are unfavorable or unpleasant for the subject [1]. For example, in situations where individuals come to perceive that their lives are not going well in a desirable direction as a result of having followed their parents’ wishes, *urami* may arise with the parents as its object [1]. English words that can correspond to *urami* include “resentment,” “grudge,” “ill feeling,” and “malice.” Independent Japanese studies on *urami* [2] reveal that the word is similar to anger in that it is an other-oriented negative emotion. According to Yamane [3], *urami* is experienced when the object of anger is directed at the assailant, and the situation in which one has been victimized is emphasized. Another discussion suggested that anger that cannot be expressed openly and remains within the individual may shift into *urami* [4]. Thus, it can be considered a subcomponent of anger. Actually, when participants were asked to describe what *urami* feels like, 13% of participants used the word “anger” to explain it [2]. However, although less frequent than anger, emotion words such as sadness (3%) and disgust (1%) also appeared in the description [2]. As the relationships between *urami* and multiple basic emotions have been pointed out, *urami* can be understood as a higher-order emotion rather than a subcomponent of anger. Based on this premise, we sought to understand the emotional state that the word *urami* refers to, along with its characteristics and similarities and differences with anger. Studies on specific emotions necessitate the understanding of the concept and context in which the emotion is experienced [2,5,6]. Thus, in this study, we examined both the conceptual structure and the situations in which *urami* occurs.

Importantly, *urami* serves as a background factor in aggressive behavior. In 2025, “*urami* due to unfulfilled feelings of affection” accounted for approximately 20% of the motives of perpetrators in stalking cases [7]. Interestingly, although *urami* is an emotion directed at a specific person, when it is externalized in the form of aggressive behavior, the target is often a large, unspecified group of people unrelated to that specific person. Approximately 20% of the motives behind indiscriminate killing incidents were *urami* toward specific individuals [8]. This may be because *urami* is a frustration that “remains inside” [4], arising only after ill treatment by someone who cannot easily be avenged and, therefore, cannot be directly targeted. Exploring the mechanisms and ways to reduce *urami* may not only reduce individual suffering but also contribute to ensuring the safety of many people in society. Despite its social importance, few empirical studies have been conducted on *urami*.

### Psychological studies on *Urami*

Systematic psychological research on *urami* has been conducted against the backdrop of interest in stalking-related behaviors. An interview study’s results defined *urami* as a psychological state in which a person (1) feels that there is nothing one can do about an unpleasant situation caused by the words of another person (helplessness), (2) believes that the words of the other person are injustice (sense of injustice), and (3) cannot forgive the assailant (unforgiveness) [1]. A “trait *urami* scale” with factors corresponding to these three components of helplessness, injustice, and unforgiveness was developed and validated [9]. Furthermore, the trait *urami* correlated with higher levels of rumination and difficulties in emotion regulation through reappraisal [10]. However, studies using this scale are still limited, and the examination of its components and scale development were conducted based on a small sample size of 14 [1]. Therefore, it is necessary to examine the generalizability of the conceptual structure through the present study.

In contrast, Suzuki [2] asked participants to freely describe the concept and context of *urami*, attempting to examine the conceptual structure and situations involving *urami* through text mining. Text mining for several hundred samples ensures a certain extent of generalizability and captures the detailed responses of participants [11]. However, no studies have used such several hundred text data to examine the relationship between *urami* experience and individual differences among participants, as well as closely related concepts such as anger. Thus, we applied structural topic modeling (STM) [12], a novel algorithm in natural language processing (NLP), to precisely understand the concept of *urami* and the situations involving *it* by quantifying text data and the relationships with other variables.

### Individual traits that can characterize *Urami* experiences

In addition to the frequency of *urami* experiences [2,9], the situations in which individuals are particularly likely to experience *urami* are also important indicators for evaluating individual differences. Examining situations in which participants are likely to experience *urami* based on their individual traits may provide valuable insights into the risks of stalking and other forms of aggressive behavior. In this study, we focused on the following five individual traits that may be related to *urami* and examined the correlations between each scale score and the descriptions of situations involving *urami*.

The first trait was interoceptive awareness. Interoception is the sensations that arise from within one’s body [13,14]. The importance and perception of physical reactions in emotional experiences and emotion regulation have been previously described [15]. A hypothetical model reported that excessive perception of noise in physical reactions results in persistent negative emotions, such as depression and anxiety [16], and that individuals who can accurately perceive their own heartbeat can appropriately control their emotions when they experience social exclusion [17]. Based on the findings regarding the relationship between interoceptive awareness and negative emotions in social situations, it is believed that indicators such as the inaccuracy of interoception and excessive attention to interoception are related to the experience of *urami*.

The second trait was narcissism. Stalking perpetrators are often diagnosed with Group B personality disorders, which are characterized by emotional and behavioral instability, including narcissism [18,19]. Perpetrators’ narcissistic personality disorder tendencies are particularly strongly correlated with the desire to threaten victims [20]. Traditional theories of narcissism have emphasized its oblivious aspect (characterized by a lack of concern for others [21]), however, in recent years narcissism has been understood as a two-dimensional concept that also includes a hypervigilant aspect (characterized by sensitivity to evaluation from others [22]). Although many unknowns are involved in the mechanisms behind this relationship, the greater the oblivious narcissistic tendency or the hypervigilant narcissistic tendency, the more likely it is that a person will feel *urami* toward the opponent, which may trigger stalking. A high level of narcissism is particularly likely to be related to *urami* in romantic relationships.

The third, fourth, and fifth traits were motivation for revenge, forgiveness, and self-efficacy, respectively. Motivation for revenge is the drive to eliminate the person who has caused one harm [23,24]. Forgiveness is treated as the opposite of *urami*, resentment, and grudges [25,26]. Self-efficacy refers to “people’s judgments of their capabilities to organize and execute courses of action required to attain designated types of performances” [27]. In particular, the term “generalized self-efficacy” refers to behavior in more generalized, everyday situations over the long term [24]. These three individual traits or scales were used to validate the trait *urami* scale [6]. These three concepts respectively correspond to the components of urami (helplessness, injustice, and unforgiveness) [1,9]. The scores of the trait *urami* scale positively correlated with the motivation for revenge and negatively correlate with forgiveness tendencies and self-efficacy [9].

### STM

To conduct an effective analysis of text data, we used STM [12]. Topic modeling is a general term for NLP algorithms that identify “topics” that characterize documents. In latent Dirichlet allocation, which is the primary method used, documents are considered to be composed of multiple topics [28,29]. As topics are expressed as clusters of words that co-occur within a document, the proportion of each topic contained in that document can be calculated while simultaneously extracting each topic. A major feature of the STM, which is an advanced version of this, is the ability to incorporate metadata (external variables) linked to documents into the analysis as topical prevalence covariates, reflecting the ease with which topics appear [12]. In STM, it is possible to directly model the relationship between metadata and topics.

Although only a few studies have used STM in psychology and related fields, some of them have attempted to explore the nature of concepts based on text data obtained from participants. For example, relationships between a topic and rating scale scores regarding “awe” [30], thoughts about driverless vehicles, and individual traits such as the desire for independence and locus of control [31], as well as the distinguishability between generosity and fairness and the component of “virtue” [32], have been examined by STM. Several other psychological studies have also used STM to examine the relationship between writer attributes and topics [33,34].

When asking people to write freely about their emotions or thoughts, as done in this study, the content of their responses may be influenced by their verbalization skills. However, it is possible to examine this influence by considering each individual’s verbalization skills as a prevalent covariate. As it is possible to relate free-description data, which contain rich information, to quantitative variables, STM is an attractive method for researchers in the fields of emotion and cognition. By using STM, researchers can manage large amounts of descriptive data objectively and quantitatively.

### Present study

This study examined the following: (1) the conceptual structure of and situations involving *urami*, (2) the relationship between *urami* situations and individual traits, and (3) the discriminability between *urami* and anger through analyses of text data. As this study is positioned as exploratory research to grasp the characteristics of *urami*, no specific hypothesis was set. The results were expected to correspond to the abovementioned aims using STM. First, based on the idea that *urami* is a higher-order emotion with multiple factors, we predicted that multiple interpretable topics reflecting these factors would be extracted from the data describing the concept of and situations involving *urami*. Second, we predicted that the proportion of the extracted topics of *urami* would correlate with each rating scale score. Third, we predicted that topics specific to *urami* would be extracted by comparing the described contents of *urami* and anger.

## Materials and Methods

This study was approved by the Ethics Committee of the Graduate School of Education, Kyoto University (July 4th, 2024/CPE-649). Informed consent was obtained from all participants electronically via an online form.

### Participants

We conducted an online survey of 560 people using CrowdWorks, a crowdsourcing service (https://crowdworks.jp). People who speak Japanese as their native language and have experienced feelings of *urami* and anger were recruited to receive valid responses. Whether or not they met these criteria was self-reported. There were no other inclusion criteria. The sample size was determined for the extraction of topics using STM, the main analysis of this study. This study’s sample size was determined based on a previous study that obtained results with sufficient validity for a sample size of approximately 500 individuals [30]. The target sample size was 500, but there was a worker who used 50 accounts to receive rewards fraudulently, resulting in a non-negligible number of missing data. Additional recruitment was carried out to ensure the sample size was as originally planned. After excluding those who provided duplicate responses, did not pass the attention check, and provided free-description data obviously unrelated to *urami* or anger, the data of 489 participants (males: 247, females: 237; do not want to answer: 5; mean age: 42.32, standard deviation [SD]: 10.08; age range: 19–72) were analyzed. The participants were paid 400 yen as monetary compensation via CrowdWorks.

### Open-ended items

#### Concepts.

For the items pertaining to the concept, we asked the participants to describe what each emotion is like in at least 40 characters, in response to the questions “What is *urami*?” and “What is anger?” for the *urami* and anger conditions, respectively. The instructions used in the study have been included in the S1 Table in the appendix. This is a commonly used instruction to examine recognition of concepts, and has been used in research on “*urami*,” “emptiness,” and “loneliness” [2,5,6]. For each question, there were 1–3 answer boxes, and these were combined for analyses. Moreover, we sought to determine subjective distinguishability (to what extent participants distinguished between the two emotions in daily life) by using an 8-point Likert scale.

#### Situations.

In the items pertaining to the situation, we asked the participants to describe in at least 40 characters the event that triggered *urami* in the *urami* condition and anger in the anger condition. The instructions were the same as those used in a previous study that required free descriptions of the situations in which *urami* occurred [2]. The instructions used in the study have been included in the S1 Table in the appendix. Additionally, participants were asked to report how many years ago the event had occurred. If it was less than a year ago, they were instructed to respond using decimal values.

#### Physical sensations.

Based on the previous findings that different emotional states can be discriminated by subjective physical sensations and neural representations that arise in response to stimuli [35–37], we also investigated the physical sensations that arise when experiencing *urami* and anger. The instructions used in the study have been included in the S1 Table in the appendix. As not all participants necessarily experienced physical sensations associated with these emotions, responses were optional, and no minimum character count was set. Because STM was not performed due to the limitations of the data amount, the results for this item were not reported in this article.

### Scales

#### IAS.

As the first interoceptive awareness measure, the IAS [37] (Japanese version: [38]) was used to assess beliefs about how accurately individuals perceive their own interoceptive sensations. Each item was presented as a single sentence, “I can always accurately perceive when ***,” with different physical sensations inserted into the blank space. For example, “my heart is beating fast (item 1)”, “I am hungry (item 2)”, and “I am breathing fast (item 3)” are inserted into ***. Participants responded to 21 items using a 5-point Likert scale: (1) strongly disagree, (2) disagree, (3) neutral, (4) agree, and (5) strongly agree. Following the recommendations of the authors of the Japanese version [38], 20 items were used for the analysis, excluding item 7 due to its low factor loading. The Cronbach’s alpha coefficients for internal consistency in the Japanese validation study [38] and this study were .91 and .91, respectively. All other numbers of items and subscales were the same as in the original version [37]. Higher scores indicate stronger beliefs in the accuracy of one’s interoceptive sensations.

#### IATS.

The IATS [39] (Japanese version: [38]) was used to assess the extent to which individuals directed their attention to interoceptive signals. Each item was presented as a single sentence, “Most of the time, my attention is focused on whether ***,” with different bodily sensations inserted into the blank space. For example, “my heart is beating fast (item 1)”, “I am hungry (item 2)”, and “I am breathing fast (item 3)” are inserted into ***. Participants responded to 21 items using a 5-point Likert scale: (1) strongly disagree, (2) disagree, (3) neutral, (4) agree, and (5) strongly agree. For the same reason as with the IAS, 20 items were used for analysis, excluding item 7. The Cronbach’s alpha in the Japanese validation study [38] and this study were .93 and .93, respectively. All other numbers of items and subscales were the same as in the original version [39]. Higher scores indicate greater tendency to pay attention to interoceptive signals.

#### The Hypervigilant-Oblivious Narcissism Scale.

The Hypervigilant-Oblivious Narcissism Scale [40] was adopted to examine separately the relationships between the two aspects included in dominant categorization of narcissism [22,40] and *urami* experience. It comprises two subscales: oblivious narcissism (10 items; e.g., “I have an innate, exceptional talent”) and hypervigilant narcissism (eight items; e.g., “I become extremely upset when my decisions or failures are criticized even slightly”). Participants responded to 18 items using a 5-point Likert scale: (1) does not apply, (2) applies somewhat, (3) is neutral, (4) applies moderately, and (5) applies completely. The Cronbach’s alpha of oblivious narcissism subscale in the development study [40] and this study were .91 and .80, respectively. The Cronbach’s alpha of hypervigilant narcissism subscale were .89 and .85, respectively. The mean score was calculated for two subscales. Higher scores indicate higher levels of narcissistic traits in each aspect.

#### “Revenge” subscale of the transgression-related interpersonal motivations inventory.

The Transgression-Related Interpersonal Motivations Inventory measures the motivation for revenge [23] (Japanese version: [41]). The Japanese version’s “revenge” subscale comprises five items, such as “I’m going to get even.” Participants responded using a 5-point Likert scale: (1) strongly disagree, (2) disagree, (3) neutral, (4) agree, and (5) strongly agree. The Cronbach’s alpha in the Japanese validation study [41] and this study were .89 and .87, respectively. The factor structure and number of items were the same in the original [23] and Japanese versions [41]. Higher scores indicate greater motivation for revenge.

#### “Forgiveness” subscale of the forgiveness of others scale.

The Forgiveness of Others Scale measures the tendency for forgiveness [42]. Its “forgiveness” subscale comprises 10 items, such as “If the other person asks for forgiveness, I would forgive them.” Participants responded using a 4-point Likert scale: (1) does not apply, (2) somewhat applies, (3) applies, and (4) strongly applies. The Cronbach’s alpha coefficients for internal consistency in the development study [42] and this study were .88 and .79, respectively. Higher scores indicate greater forgiveness tendency.

#### The scale measuring a sense of generalized self-efficacy.

The Scale Measuring a Sense of Generalized Self-Efficacy measures generalized self-efficacy [43]. It comprises seven items, such as “I feel that I can usually do most things without much effort.” Participants responded using a 5-point Likert scale: (1) strongly disagree, (2) disagree, (3) neutral, (4) agree, and (5) strongly agree. The Cronbach’s alpha in the development study [43] and this study were .92 and .80, respectively. Higher scores indicate greater trait self-efficacy.

#### “Describing” subscale of the five facet mindfulness questionnaire.

As the free descriptions of emotional states can be influenced by verbalization skills, the Five Facet Mindfulness Questionnaire (FFMQ-d) [44,45] was incorporated into the STM as a prevalence covariate (also as a control variable). Its Japanese version [46] comprises five items, such as “I’m good at finding words to describe my feelings.” Participants responded using a 5-point Likert scale: (1) never or very rarely true, (2) rarely true, (3) sometimes true, (4) often true, and (5) very often or always true. The Cronbach’s alpha in the Japanese validation study [46] and this study were .91 and .76, respectively. The factor structure and number of items were the same in the original [45] and Japanese versions [46]. Higher scores indicate greater verbalization skills.

### Procedure

The survey was created and conducted using an online survey system (Qualtrics, https://www.qualtrics.com). Participants read the instructions regarding the survey, and only those who provided written informed consent answered all the questions. The recruitment and data collection for the survey were conducted from August 22 to August 29, 2024. At the beginning of the survey, we asked the participants to provide their age and gender. Participants could choose “male”, “female”, “other”, and “do not want to answer.” They then responded to free-description items pertaining to the concepts, situations, and physical sensations related to *urami* and anger. Additionally, they answered the seven abovementioned rating scales. The order in which the emotional conditions and scales were presented was randomized for each participant. In the Interoceptive Accuracy Scale (IAS) and the Interoceptive Attention Scale (IATS), an item stating, “For this item, please select (1) ‘strongly disagree’” was inserted as an attention check. The survey took approximately 25 minutes.

### Data analyses

The main analysis was conducted using the statistical software R [47], following the approach of [30]. The RMeCab package [48] was used to preprocess the Japanese text data. The docDF() function was employed to extract parts of speech that carried interpretable meanings (verbs, nouns, adjectives, and adverbs), and the inflected words were unified into their root forms. Additionally, words that could become noise owing to their frequent appearance in various contexts (such as “する [do],” “なる [become],” “ない [not],” “いる [be],” and “ある [exist]”) were removed as stop words.

STM was performed using the “stm” package [49]. Consistent with prior recommendations [49], spectral initialization was used for model initialization. A default cutoff value of 500 was used to terminate the EM (Expectation–Maximization) iterations when the model did not converge. The procedure for determining the total number of topics (K) was explored by following the guidelines and using the searchK() function [50]. The range of *K* explored covered all integers between 3 and 20. For all these models, four metrics (semantic coherence, exclusivity, variational lower bound, and residuals) were plotted to evaluate the overall quality of the topics. The optimal number of topics for each analysis was determined based on these metrics. The topic-naming procedure also followed the guidelines of Weston et al. [50]. The first method for naming topics involved checking the words with high occurrence probabilities (*β*) for each topic, and the second method involved examining the content of documents with high topic proportions (*θ*) for each topic. For each topic, the words with high occurrence probabilities and documents with high topic proportions were output and the topics were named by reviewing these words and descriptions.

Two-stage STM analyses were performed on text data of concepts and situations. The first stage of STM addressed the first research aim and focused solely on the text data related to *urami*. In the STM analysis of the concept of *urami*, FFMQ-d was included as a prevalence covariate. In the STM analysis of the situations involving *urami*, in addition to age and the FFMQ-d, all scale scores were included as prevalence covariates to examine their relationships (as described later).

Next, regression analysis was conducted using the estimateEffect() function, with scale scores as independent variables and topic proportions obtained from the STM of the situations involving *urami* as dependent variables. As the focus was on the direct correlational relationships between each scale score and topic proportions, simple regression models were used to examine how each scale score predicted the topic proportions. In this analysis, the Benjamini-Hochberg procedure [51] was used with a false discovery rate (FDR) of 0.05 to account for the increased risk of Type I error due to multiple testing. This analysis addressed the second research aim.

In the second stage of the STM, *urami* and anger text data regarding concepts and situations were analyzed. The model included described emotions (*urami* or anger), age, FFMQ-d scores, and subjective distinguishability (degree to which *urami* and anger are subjectively distinguished). Age, FFMQ-d scores and subjective distinguishability were treated as control variables. The estimateEffect() function was used to examine the effects of these three variables. FDR correction [51] with the same conditions as above was applied.

Thus, this study examined four topic models corresponding to each research aim: the concept of *urami* (aim 1), situations involving *urami* (aims 1 and 2), the concept of *urami* and anger (aim 3), and situations involving *urami* and anger (aim 3).

## Results

### Components of *Urami*

**Fig 1:**
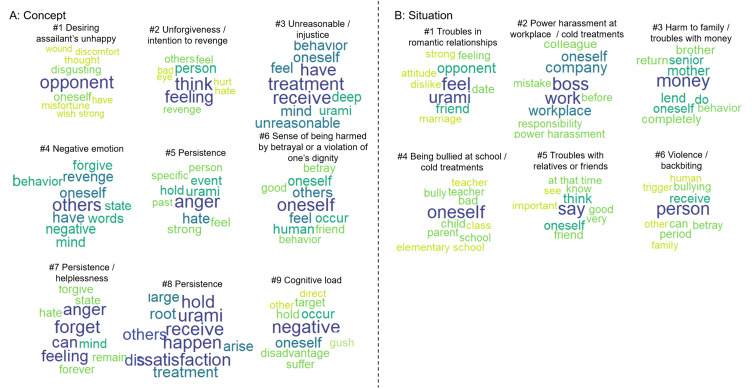
Words with high occurrence probabilities for each topic. The original Japanese word list was translated into English, and a word cloud was generated. As “emotion (感情)” was assigned to many topics, it was excluded from the word cloud to avoid hindering the interpretation of the topics. Panels A and B represent the STM results for the concept of and situations involving *urami*, respectively. The closer the hue is to blue, the higher the occurrence probabilities.

For the text data regarding the concept of *urami*, after reviewing the four indicators (semantic coherence, exclusivity, variational lower bound, and residuals), the optimal number of topics was determined to be nine. Plots of each indicator are depicted in S1 Fig-A in the appendix. The naming results for the nine topics identified by STM, as well as the words with high occurrence probabilities (*β*) for each topic, are presented in Fig 1-A and S2 Fig-A. The documents with high topic proportions (*θ*) for each topic are listed in S2 Table in the appendix.

The FDR correction was applied to 18 tests based on the combination of nine topics and two control variables (i.e., age and FFMQ-d score). Age positively predicted the proportion of Topic 3 (unreasonable / injustice; *b* = 0.002, *SE* = 0.00, *t* = 2.85, *p* = .030), and negatively predicted the proportion of 4 (negative emotion; *b* = −0.002, *SE* = 0.00, *t* = −2.66, *p* = .036) and 5 (persistence; *b* = −0.003, *SE* = 0.00, *t* = −3.61, *p* < .05). These results indicated that Topic 3 (unreasonable / injustice) was more likely to be discussed by older participants, whereas Topic 4 (negative emotion) and 5 (persistence) were more likely to be discussed by younger participants. The FFMQ-d score negatively predicted the proportion of Topic 3 (negative emotion; *b* = −0.03, *SE* = 0.01, *t* = −2.81, *p* = .030). The effec*t*s of these variables in other topics did not survive the FDR correction (*p*s > .050).

### Situations involving *Urami*

For the text data regarding situations involving *urami*, based on the indicators (S1 Fig-B in the appendix), six topics were determined to be the optimal number. Fig 1-B and S2 Fig-B presents the naming results for each topic identified by STM, along with words that have high occurrence probabilities (*β*) for each topic. S3 Table in the appendix lists the documents with high topic proportions (*θ*) for each topic. The effects of control variables were mentioned in the next section.

### Relationships with rating scale scores

Table 1 presents the descriptive statistics for each scale score. The results of the regression analysis, with topic proportions related to the situations involving *urami* as the dependent variable, are presented in S4 Table in the appendix. The FDR correction was applied to 54 tests based on the combination of six topics and nine covariates. Regarding control variables, age positively predicted the proportion of Topic 3 (harm to family / troubles with money) and negatively predicted the proportion of Topic 4 (being bullied at school / cold treatments). The FFMQ-d score positively predicted the proportion of Topic 3 (harm to family / troubles with money).

**Table 1:** Mean Score and Standard Deviation (SD) of each rating scale.

Scale	*Mean*	*SD*
Interoceptive Accuracy Scale	3.60	0.62
Interoceptive Attention Scale	2.64	0.74
Hypervigilant-oblivious narcissism scale		
Oblivious narcissism	2.41	0.80
Hypervigilant narcissism	2.98	0.89
Transgression-Related interpersonal Motivations Inventory		
Revenge	3.24	0.96
Forgiveness of Others Scale		
Forgiveness	1.76	0.52
Scale Measuring a Sense of Generalized Self-Efficacy	2.70	0.93
Five Facet mindfulness Questionnaire		
Describing	2.82	0.89

Regarding target variables, interoceptive attention positively predicted the proportion of Topic 6 (violence / back biting). Hypervigilant narcissism positively predicted the proportion of Topic 6 (violence / back biting) and negatively predicted the proportion of Topic 3 (harm to family / troubles with money). Motivation for revenge negatively predicted the proportion of Topic 3 (harm to family / troubles with money). Self-efficacy negatively predicted the proportion of Topic 6 (violence / back biting).The effects of other variables did not survive the FDR correction (*corrected p*s > .050).

### Distinctiveness in components

For the text data on the concepts of *urami* and anger, the optimal number of topics was determined to be 11 (see S3 Fig-A in the appendix for each indicator). The FDR correction was applied to 44 tests based on the combination of 11 topics and four covariates (i.e., emotional condition, age, FFMQ-d score, and subjective distinguishability).

**Fig 2:**
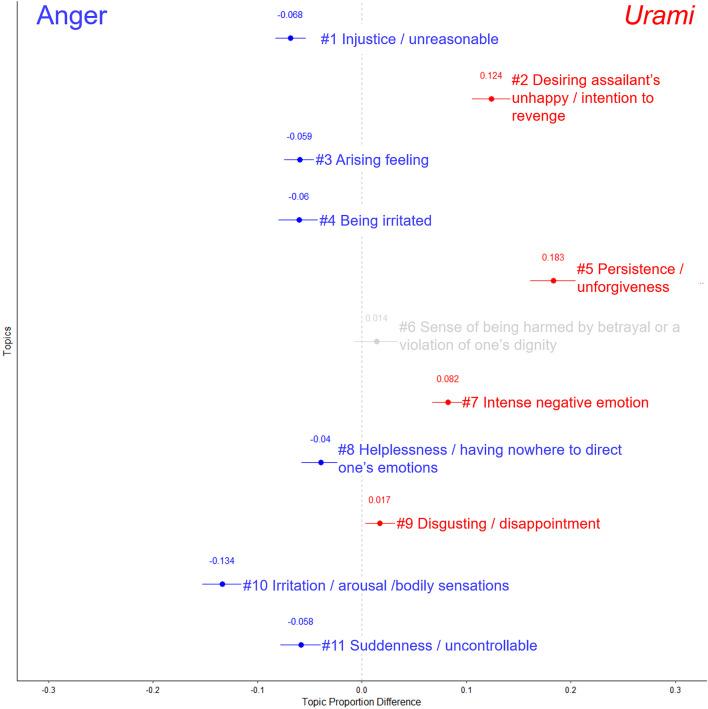
Naming results for each topic related to the concepts of *urami* and anger, and the effects of emotional conditions. The points plotted for each topic represent the regression coefficients and the error bars depict the 95% confidence intervals. Topics that significantly appeared more frequently when participants were asked to describe *urami* and anger are marked in red and blue, respectively. The light blue and red colors indicate that the test results did not survive the FDR correction.

Based on the descriptions with high topic proportions *θ* identified by STM (presented in S5 Table in the appendix), Fig 2 shows the names of the topics and their corresponding emotional condition effects. S4 Fig-A and S5 Fig-A in the appendix shows the words with the highest occurrence probabilities for each topic. In the texts describing *urami*, Topic 2 (desiring assailant’s unhappy / intention to revenge; *b* = 0.12, *SE* = 0.01, *t* = 13.24, *p* < .001), 5 (persistence / unforgiveness; *b* = 0.18, *SE* = 0.01, *t* = 17.86, *p* < .001), 7 (in*t*ense negative emotion; *b* = 0.08, *SE* = 0.01, *t* = 9.32, *p* < .001), and 9 (disgusting / disappoin*t*ment; *b* = 0.02, *SE* = 0.01, *t* = 2.48, *p* = .041) appeared more frequently. In contras*t*, in the texts describing anger, Topic 1 (injustice / unreasonable; *b* = −0.07, *SE* = 0.01, *t* = −8.95, *p* < .001), 3 (arising feeling; *b* = −0.06, *SE* = 0.01, *t* = −8.24, *p* < .001), 4 (being irrita*t*ed; *b* = −0.06, *SE* = 0.01, *t* = −6.58, *p* < .001), 8 (helplessness / having nowhere *t*o direct one’s emotions; *b* = −0.04, *SE* = 0.01, *t* = −4.79, *p* < .001), 10 (irri*t*ation / arousal / bodily sensations; *b* = −0.13, *SE* = 0.01, *t* = −13.35, *p* < .001), 11 (suddenness / uncontrollable; *b* = −0.06, *SE* = 0.01, *t* = −6.49, *p* < .001) appeared more frequently.

Regarding control variables, age positively predicted the proportion of Topic 6 (sense of being harmed by betrayal or a violation of one’s dignity; *b* = 0.002, *SE* = 0.001, *t* = 2.89, *p* = .015), and negatively predicted the proportion of Topic 4 (being irritated; *b* = −0.001, *SE* = 0.0004, *t* = −3.14, *p* = .008) and 9 (disgusting / disappointment; *b* = −0.001, *SE* = 0.0003, *t* = −2.65, *p* = .027). The FFMQ-d score negatively predicted the proportion of Topic 8 (helplessness / having nowhere to direct one’s emotions; *b* = −0.02, *SE* = 0.004, *t* = −4.39, *p* < .001). The effects of other covariates including subjective distinguishability did not survive the FDR correction (*p*s > .050).

### Distinctiveness in situations

For the text data on the situations involving *urami* and anger, the optimal number of topics was determined to be 11 (see S3 Fig-B in the appendix for each indicator). The FDR correction was applied to 44 tests based on the combination of 11 topics and four covariates.

**Fig 3:**
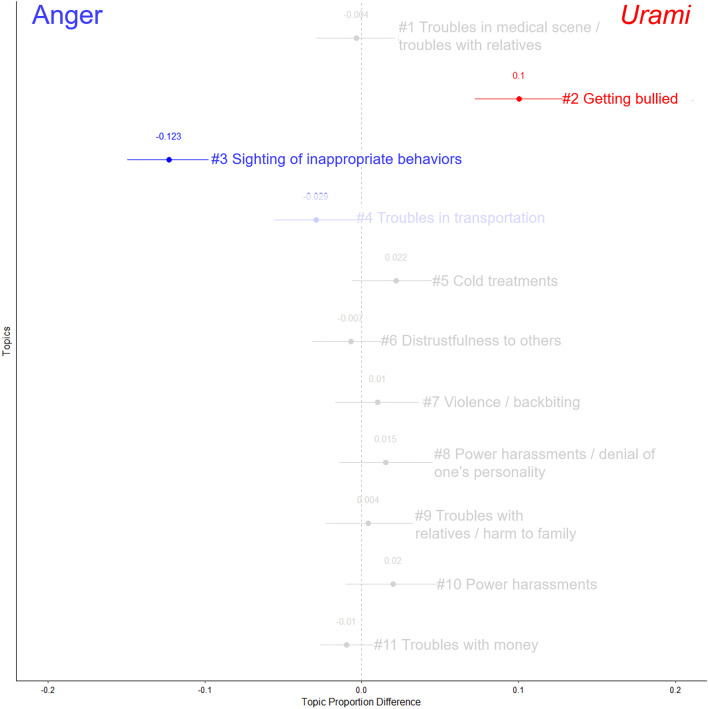
Naming results for each topic related to the occurrence situations of *urami* and anger, and the effects of emotional conditions. The points plotted for each topic represent the regression coefficients and the error bars depict the 95% confidence intervals. Topics that significantly appeared more frequently when participants were asked to describe urami and anger are marked in red and blue, respectively. The light blue and red colors indicate that the test results did not survive the FDR correction.

Considering the topics identified through STM, topic names were determined based on the content with the highest topic proportions (S6 Table in the appendix). Fig 3 illustrates the influence of emotional conditions on each topic. Additionally, S4 Fig-B and S5 Fig-B in the appendix shows the words with the highest occurrence probabilities for each topic. In the texts describing *urami*, Topic 2 (getting bullied; *b* = 0.10, *SE* = 0.02, *t* = 6.58, *p* < .001) appeared more frequently. In contrast, in the texts describing anger, Topic 3 (sighting of inappropriate behaviors; *b* = −0.12, *SE* = 0.01, *t* = −9.03, *p* < .001) appeared more frequen*t*ly.

Regarding control variables, age negatively predicted the proportion of Topic 2 (; *b* = −0.004, *SE* = 0.001, *t* = −5.30, *p* < .001). The effects of other covariates did not survive the FDR correction (*p*s > .050).

The results of the paired t-test indicate that the time when the event that triggered the feeling of *urami* occurred (mean 13.37 years ago) was significantly earlier than the time when anger occurred (mean 8.58 years ago; *t*(488) = 9.01, *p* < .001, *d* = 0.41).

## Discussion

This study examined the conceptual structure and related situations of *urami* using STM, the method to quantify relationships between text and metadata. The results showed that *urami* is a persistent emotion, and is likely to arise from experiences such as bullying at school. They also suggested that *urami* can be distinguished from anger, a closely related concept in these aspects. Furthermore, levels of personality traits such as hypervigilant narcissism were associated with a greater tendency to experience *urami* in specific situations.

### Components of and situations involving *Urami*

The topic modeling extracted meaningful topics that are consistent with previous study that identified “injustice”, “unforgiveness” and “helplessness” as components of *urami* [1,6]. Among the topics identified from the descriptions regarding the concept of *urami*, “unreasonable / injustice” corresponds to “injustice,” while “desiring assailant’s unhappy” and “unforgiveness / intention to revenge” correspond to “unforgiveness.” In this study, the topics corresponding to “unforgiveness” were subdivided, and two new topics were identified. Topics named “persistence / helplessness” is thought to correspond to “helplessness”. Interestingly, the number of topics named “persistence” was emphasized. “Unforgiveness”, although labeled differently, may also include such elements of persistence. From the perspective of the persistence of emotion [52], two distinct patterns emerged: one involving the motivation for revenge against the perpetrator and another focused solely on desiring the perpetrator’s unhappiness. The newly identified “negative affect” reflects emotional valence, and “sense of being harmed” reflects the condition of occurrence, both of which can be interpreted as topics representing fundamental elements that constitute emotion. Additionally, these topics demonstrate consistency with previous definitions and indicate the existence of multiple subtypes of *urami* that differ in their other-oriented nature. “Cognitive load” reflects those who mentioning that the persistence of *urami* strains cognitive resources, suggesting that *urami* may reduce well-being. Unresolved events tend to come to mind even during unrelated tasks [53], and this topic is considered to reflect such a phenomenon.

Interpretation for the effects of control (non-target) variables are unpredicted and tentative. However, older participants tended to report Topic 3 (unreasonable / injustice) as concept of *urami*, whereas younger participants reported Topics 4 (negative emotion) and 5 (persistence). This may suggest that older participants are more likely to refer to higher-order concepts. Lower verbalization skills (FFMQ-d scores) were related to the prevalence of Topic 3, which may reflect that perceived injustice is a central component of *urami* that can be recognized even by those with lower verbalization skill.

The topics pertaining to situations involving *urami* indicate that *urami* arises in various settings, such as romantic relationships, workplaces, and schools. This aligns with findings from a prior study [2] that examined the occurrence of *urami* using similar procedures. Bullying experienced in these situations has been shown to cause significant damage to quality of life [54], and the persistent negative emotional state it triggers may be labeled *urami*. Furthermore, events that triggered *urami* tended to have occurred further in the past than those that triggered anger. Although these events were less frequent, their greater impact on life may have made them more vivid.

### Relationships with individual traits

The analysis of the relationship between psychological scale scores and situations involving *urami* indicates that the likelihood of experiencing *urami* varies depending on certain individual traits. Participants with low levels of motivation for revenge tended to experience *urami* in the situations described in Topic 3 (harm to family / troubles with money). Topic 3 was also associated with lower levels of hypervigilant narcissism. In cases where the violation of one’s own dignity is minor—such as harm directed at a family member—people may be (relatively) less likely to feel a motivation to revenge.

Individuals with higher hypervigilant narcissism and lower self-efficacy were more likely to experience *urami* related to Topic 6 (violence backbiting). Individuals with high levels of hypervigilant narcissism, who are more sensitive to others’ evaluation [22,40], may be more prone to feel unpleasant when their self-image is threatened by violence and backbiting from others. This can lead to a stronger association with *urami*. It is interesting that individuals who pay greater attention to their internal bodily states (i.e., interoceptive signals) were more likely to feel *urami* in response to violence.

Regarding control variables, older participants showed higher prevalence of Topic 3 (harm to family / troubles with money), whereas younger participants showed higher prevalence of Topic 4 (being bullied at school / cold treatments). If individuals had multiple experiences of *urami*, this may be because more recent events were more likely to be reported. The association between verbalization skills and Topic 3 is difficult to interpret. However, as noted above, individuals with higher verbalization skills may be able to appropriately describe situations even when the violation of their own dignity is relatively minor.

The lack of significant effects for most variables may be due to the use of topic proportions as the dependent variable, rather than the intensity or frequency of emotional experiences. To the best of our knowledge, there are no studies that have examined the relationship between emotional triggers and individual traits using similar approach, which makes these interpretations tentative. Future research should experimentally manipulate situations and measure the intensity of *urami* elicited in each context to further investigate the relationship between *urami* experiences and individual differences.

### Differences from anger

The analysis including emotional condition as a prevalence covariate showed that *urami* and anger differ in their components. When participants were asked to describe the concept of *urami*, topics related to persistence—such as “desiring assailant’s unhappy / intention to revenge”—were discussed more frequently. In contrast, when describing anger, topics related to “irritation / arousal / bodily sensations” or “suddenness / uncontrollable” were more prevalent. This strongly supports the claim that *urami* is an emotion characterized by persistence—a crucial factor in distinguishing and defining emotions [52,55]. A study that examined the duration of 27 different emotions reported that anger lasts for approximately 24 hours on average [55]. If a similar procedure were used to assess the duration of *urami*, it would likely be reported as a longer-lasting emotion, similar to hatred or sadness. Among the components of *urami* [9], injustice and helplessness appeared more frequently in anger. For these to constitute components of *urami*, they may need to be experienced persistently.

Regarding the control variables, as with the concepts of *urami*, younger participants tended to report relatively simple concepts such as Topics 4 (being irritated) and 9 (disgusting / disappointment), whereas older participants tended to report more complex concepts such as Topic 6 (sense of being harmed by betrayal or a violation of one’s dignity). Lower verbalization skills were associated with Topic 8 (helplessness/ having nowhere to direct one’s emotions), which may be because the content of Topic 8 reflects a central component of resentment that can be reported even by individuals with lower verbal ability. However, note that this interpretation is also tentative.

Unlike conceptual differences, only a few topics differed in prevalence between *urami* and anger in the situation. When participants were asked to describe situations involving *urami*, Topic 2 (getting bullied) was discussed more frequently. In contrast, when describing anger, Topic 3 (sighting of inappropriate behaviors) was discussed more frequently. Overall, *urami* tended to occur in situations where individuals were harmed by people with whom they had close or frequent interactions, such as classmates, and colleagues. In contrast, anger was more likely to arise in temporary relationships with less personal significance. When a perpetrator is close to someone, severing ties is often difficult. Continuing a relationship with the perpetrator after the harm has occurred may contribute to the persistence of negative emotions, ultimately leading to *urami*. Among the situational factors that influence emotion elicitation [56], anger has been suggested to arise when the perceived ability to change a situation is relatively low [57]. *Urami* may be associated with an even greater perceived helplessness compared to anger.

In both conceptual and situational analyses, some topics appeared with a similar frequency, regardless of whether the described emotion was *urami* or anger. This reflects a conceptual overlap between the two emotions, supporting the idea that *urami* is a higher-order emotion containing both unique components and components shared with anger.

Taking advantage of the strengths of STM [12], this study incorporated several control variables and quantified the magnitude of their effects. It was assumed that individual description skills may influence free descriptions; however, contrary to this assumption, their effect was not significant for all topics. Although we only recruited participants who had experienced *urami* toward others, it is possible that *urami* is a psychological state that is relatively easy to verbalize. Similarly, the effect of subjective distinguishability was not observed in any topics, suggesting that, even if individuals do not consciously differentiate between the terms *urami* and anger, they may still internally represent them as distinct emotional concepts. However, subjective distinguishability was measured with a single-item scale that is general and providing limited information; therefore, there might be a limitation to its interpretation.

### Social implications

This study can be positioned as an initial approach toward addressing social issues related to *urami*. *Urami* may underlie various aggressive behaviors, such as stalking and homicide, and is likely rooted in the distress of persistent unforgiveness and perceived unfairness. While individual factors such as narcissism and self-efficacy are related, situational factors such as being bullied at school are also important and cannot be overlooked. Demonstrating this quantitatively using STM is a key contribution of this study, and it may provide evidence for social interventions aimed at preventing *urami*, such as education.

### Limitations and future prospects

A key contribution of this study is its multifaceted approach to the concept of *urami*. However, some limitations must be considered. First, the naming of topics is inherently constrained by methodological factors, as it relies on researchers interpreting and labeling each topic based on individual descriptions.

Second, because this study analyzed free-description data, it is possible that only participants who were highly skilled at describing their thoughts were included. Although the self-reported describing skill measured by the FFMQ-d was only weakly associated with topic frequency and showed no extreme deviations, it remains to be seen whether similar results would be obtained using objective linguistic measures, such as vocabulary size. Regardless, developing a new psychological scale to assess the traits or states of *urami* based on collected text data could help explore how it manifests as a psychological state among individuals with lower verbalization skills.

Third, the number of concepts/variables to examine relationships with *urami* was limited. The association with narcissism highlights the social-emotional aspect of *urami*. It would be interesting to extend this finding by examining its relationships with social psychological process such as empathy or self-consciousness.

Finally, although *urami* and anger were suggested to be fundamentally distinguished by their persistence, this study focused more on the conceptualization of emotions rather than on their temporal dynamics. As the next step, the chronological relationship between *urami* and anger should be examined. Specifically, negative emotions arising in similar situations may initially manifest as anger and later be recognized as *urami* after a certain period. This temporal shift may explain the observed overlap in descriptive data across conditions.

## Conclusion

This study examined the nature of *urami*, a concept rooted in Japanese culture, using a bottom-up approach with NLP. The results revealed that *urami* is a persistent emotion typically experienced in situations where harm is caused by intimate others, distinguishing it from anger. Individuals with higher levels of certain personality traits, such as narcissism, were more likely to experience *urami* in specific situations. This study is the first to explore the conceptual structure of emotions and the situations that involve them using STM, while simultaneously revealing their associations with individual traits and closely linked concepts. Expanding this research to include cross-cultural comparisons with concepts such as “resentment” or “grudge” could potentially serve as a foundation for discussing the causes and solutions to conflicts within different cultural contexts.

## Supporting information

S1 Fig Four indices used to determine the number of topics (Semantic Coherence, Exclusivity, Variational Lower Bound, Residuals). A represents the indices calculated for the concept of *urami*, while B represents those calculated for the situations of *urami*.
(TIF)

S2 Fig High-frequency words in each topic. This figure is the original Japanese version of Fig 1 in the manuscript. A represents the STM results for the concept of *urami*, while B represents the results for the situations of *urami*. The word “emotion (感情)” was excluded from the word cloud, as it was assigned to multiple topics and could hinder interpretation. The closer the hue is to blue, the higher the occurrence probability.
(TIF)

S3 Fig Four indices referenced for determining the number of topics (Semantic Coherence, Exclusivity, Variational Bound, and Residuals). A represents the indices calculated for the concepts of *urami* and anger, while B represents the indices for the situations of *urami* and anger.
(TIF)

S4 Fig Words with high occurrence probabilities for each topic. The original Japanese word list was translated into English, and a word cloud was generated. A represents the STM results for the concept of both *urami* and anger, while B represents the STM results for the situation of both *urami* and anger. Since “emotion (感情)” was assigned to many topics, it was excluded from the word cloud to avoid hindering the interpretation of the topics. The closer the hue is to blue, the higher the occurrence probability.
(TIF)

S5 Fig High-frequency words in each eopic. This figure is the original Japanese version of S4 Fig in the appendix. A represents the STM results for the concept of both *urami* and anger, while B represents the results for the situations of both *urami* and anger. The word “感情” was excluded from the word cloud, as it was assigned to multiple topics and could hinder interpretation. The closer the hue is to blue, the higher the occurrence probability.
(TIF)

S1 Table Instructions of open-ended items. (DOCX)

S2 Table Examples of documents with high topic proportion (θ) for the concept of *urami.* Excerpts were selected based on their high interpretability and ease of translation into other languages.
(DOCX)

S3 Table Examples of documents with high topic proportion (θ) for the situation of *urami.* Excerpts were selected based on their high interpretability and ease of translation into other languages.
(DOCX)

S4 Table Estimated values (b), Standard Errors (SE), and p-values for prevalence covariates in each topic regarding situation of *urami.* Bold values indicate that the effect of the prevalence covariate was statistically significant at the 5% level.
(DOCX)

S5 Table Examples of documents with high topic proportion (θ) for the concept of both *urami* and anger. Excerpts were selected based on their high interpretability and ease of translation into other languages.
(DOCX)

S6 Table Examples of documents with high topic proportion (θ) for the situation of both *urami* and anger. Excerpts were selected based on their high interpretability and ease of translation into other languages.
(DOCX)
