# Supplementary material for: The feeling of “Urami”: A structural topic modeling approach
Source: PLoS One. 2026 May 26;21(5):e0349193. doi: 10.1371/journal.pone.0349193 (PMC13210193; doi:10.1371/journal.pone.0349193)
Supplement: S1 Table — (DOCX) [file pone.0349193.s006.docx]

**S1** **Table. Instructions of open-ended items.**

|  | Original (Japanese) | English |
| --- | --- | --- |
| Concept (*urami*) | 「***うらみ***」とはどのような気持ちだと思いますか？あなた自身がうらみを感じた経験にもとづいて、「うらみとは」に続く形で自由にご記入ください。下記3つの回答欄のうち**最低1つに**ご回答ください。**辞書やブラウザなどで単語の意味を調べたりせず、ご自身のお考えのみをご回答ください。** ※回答欄1つあたり40文字以上ご記入ください。 | What do you think “***urami***” is like as a feeling? Based on your own experiences of urami, please complete the phrase “Urami is …” in your own words. Please respond in **at least one** of the three answer fields below. **Do not look up the meaning of the word in a dictionary or browser; answer only based on your own thoughts.**  Please write at least 40 characters in each response field. |
| Situation (*urami*) | **うらみ**を感じるきっかけになった出来事についてお答えください。 ※40文字以上でご回答ください。 | Please describe the event that triggered your **urami**.  Please write at least 40 characters. |
| Physical sensation (*urami*) | あなたが**うらみ**を感じたとき、どのような身体の感じがありましたか？具体性は問いませんので、身体のどの部位にどのような感覚があったかなど、ご自由にご記入ください。 ※この項目には回答しなくても次に進むことができます。 | When you felt **urami**, what kind of bodily sensations did you experience? Specificity is not required; please describe freely which parts of your body felt what kind of sensations.  You can proceed without answering this item. |
| Concept (anger) | 「***怒り***」とはどのような気持ちだと思いますか？あなた自身がうらみを感じた経験にもとづいて、「うらみとは」に続く形で自由にご記入ください。下記3つの回答欄のうち**最低1つに**ご回答ください。**辞書やブラウザなどで単語の意味を調べたりせず、ご自身のお考えのみをご回答ください。** ※回答欄1つあたり40文字以上ご記入ください。 | What do you think “***anger***” is like as a feeling? Based on your own experiences of feeling anger, please complete the phrase “Anger is …” in your own words. Please respond in **at least one** of the three answer fields below. **Do not look up the meaning of the word in a dictionary or browser; answer only based on your own thoughts.**  Please write at least 40 characters in each response field. |
| Situation (anger) | **怒り**を感じるきっかけになった出来事についてお答えください。 ※40文字以上でご回答ください。 | Please describe the event that triggered your **anger**.  Please write at least 40 characters. |
| Physical sensation (anger) | あなたが**怒り**を感じたとき、どのような身体の感じがありましたか？具体性は問いませんので、身体のどの部位にどのような感覚があったかなど、ご自由にご記入ください。 ※この項目には回答しなくても次に進むことができます。 | When you felt **anger**, what kind of bodily sensations did you experience? Specificity is not required; please describe freely which parts of your body felt what kind of sensations.  You can proceed without answering this item. |
