# Supplementary material for: The feeling of “Urami”: A structural topic modeling approach
Source: PLoS One. 2026 May 26;21(5):e0349193. doi: 10.1371/journal.pone.0349193 (PMC13210193; doi:10.1371/journal.pone.0349193)
Supplement: S2 Table — Excerpts were selected based on their high interpretability and ease of translation into other languages. (DOCX) [file pone.0349193.s007.docx]

| Topic | Topic name | Original documents | Documents translated in English |
| --- | --- | --- | --- |
| 1 | Desiring assailant’s unhappy | 相手のせいで、自分が不幸になったのと同様に、相手にも自分と同じように、あるいはそれ以上に不幸になって欲しいと思う感情。 負の連鎖を生む行動。一生消えることのない心の傷。相手がいなくなればいいのにと、心から願う思い。 相手によってもたらされた不幸が強ければ強いほど、相手にも強い不幸を望む気持ち。非生産的で何も生み出さないマイナスの感情。 | A feeling in which, because someone caused your unhappiness, you wish for them to become as unhappy as you—or even more so. It is a negative, unproductive emotion that creates a vicious cycle, leaves lasting emotional scars, and can make you sincerely wish the other person would disappear. The greater the suffering caused by that person, the stronger the desire for them to suffer as well. |
| 2 | Unforgiveness / intention to revenge | 何かのきっかけで取り返しのつかない屈辱を、他人から味あわされて後から振り返っても許せない気持ちになること | Experiencing an irreparable humiliation caused by someone else, and even when looking back later, being unable to forgive it. |
| 3 | Unreasonable / injustice | 理不尽な扱い、暴力的な扱いに対して強い憤りを感じるものです。 相手に対して強い恐怖をもち、その反動で抱くものです。 相手の理不尽、暴力的な扱いが　まわりからも同様な扱いを受けることによって増幅するものです。 | It is a strong sense of anger toward unfair or violent treatment. It can arise from a deep fear of the other person, as a kind of reaction to that fear. It may also be intensified when such unfair or abusive treatment is repeated or reinforced by others around them. |
| 4 | Negative emotion | 自分自身へのネガティブ感情というよりは、他者へ抱くネガティブな感情だと思います。 | think it is less a negative feeling toward oneself and more a negative feeling directed toward others. |
| 5 | Persistence | うらみとは、誰かのせいで失敗したり不利な立場になったことで、その誰かに対して怒りやわだかまりを持ち続けること。 | Urami is the act of continuing to hold anger and bitterness toward someone because their actions caused you to fail or put you at a disadvantage. |
| 6 | Sense of being harmed by betrayal or a violation of one’s dignity | 私自身との付き合いにおいて、表面上では常にいい顔をして非常に親密な態度をとっておきながら、裏に回ると表の顔とは真逆に悪口を言いふらしている友人の現実を知った時の感情。 | The feeling you experience when you discover that a friend who always acted kind and very close to you in public was, behind your back, spreading insults and speaking badly about you. |
| 7 | Persistence / helplessness | 怒りや憎しみのような強い気持ちが十分な時間がたっても忘れることがなく強く残っている状態 理不尽で不合理な状況が続きその理不尽さを忘れることが出来ずに長いこと心の中で悶々としている状況 理不尽な辱めを受けたがそれをした本人は忘れいるが、辱めを受けた人間は忘れることなく長いこと覚えている状態 | A state in which strong emotions like anger or hatred remain deeply even after a long time has passed. It also refers to being unable to forget prolonged unfair or unreasonable situations and continuing to brood over them. Even if the person who caused the humiliation has forgotten it, the one who suffered it remembers it for a long time. |
| 8 | Persistence | 根に持つ事。人から許せない迷惑行為をされた時に恨む。私は未だに、中学1年生の時に裏切られた事件は忘れておらず根に持ってます。 | Holding a grudge. Resenting someone when they have done something deeply hurtful or unforgivable. I still have not forgotten being betrayed in my first year of junior high school, and I still hold a grudge over it. |
| 9 | Cognitive load | 外部環境を要因とした負の事象に対して、自己の内部で負の感情を処理することが出来ずに、そのまま負の感情を抱き続ける感情。ケースによっては、負の感情の要因となった対象に対して、現実的に攻撃する場合もある。 | A feeling in which one is unable to process negative emotions caused by external events and continues to carry them within. In some cases, one may even direct real-world aggression toward the source of those emotions. |
