# Supplementary material for: The feeling of “Urami”: A structural topic modeling approach
Source: PLoS One. 2026 May 26;21(5):e0349193. doi: 10.1371/journal.pone.0349193 (PMC13210193; doi:10.1371/journal.pone.0349193)
Supplement: S3 Table — Excerpts were selected based on their high interpretability and ease of translation into other languages. (DOCX) [file pone.0349193.s008.docx]

| Topic | Topic name | Original documents | Documents translated in English |
| --- | --- | --- | --- |
| 1 | Troubles in romantic relationships | 相手の浮気によって急に恋人から別れを告げられた。その後、恋人はすぐに結婚し、結婚式の招待状まで送ってきた。 | My partner suddenly broke up with me because of their infidelity. Soon after, they got married and even sent me an invitation to their wedding. |
| 2 | Power harassment at workplace / cold treatments | 会社の業績をよくするために提案をしたら、上司から提案を否定されただけではなく、人格を否定する発言をされた。しかも、提案をしてか上司の自分に対する態度が明かに攻撃的になった | When I made a proposal to improve the company’s performance, my boss not only rejected it but also made remarks that attacked my character. Moreover, after I made the proposal, my boss’s attitude toward me clearly became more hostile. |
| 3 | Harm to family / troubles with money | 私は以前、兄の経営する企業に勤めていたことがあるのだが、兄がすぐに行動を起こさなければ大きな損失が出るような事案があったのにも関わらず、兄はパニックになりすぐに行動を起こせなかったので、私が兄の代わりに行動を起こし、大きな問題を解決したことがあった。しかし、そのことを知った兄は、勝手な行動をするなと社員の前で私を叱った。その理由としては、兄はきっと自分の経営者としての立場を守りたかったからだろう。その時の兄の口調が、あまりにもひどく、10年以上経った今でも、その時のことを恨んでいる。 | I once worked for a company run by my older brother. There was a situation that could have caused a major loss if immediate action had not been taken, but my brother panicked and was unable to act quickly. So I stepped in and handled it myself, successfully resolving the problem. However, when my brother found out, he scolded me in front of the employees for acting on my own. He probably wanted to protect his position as the manager. The way he spoke to me at that time was so harsh that, even after more than ten years, I still resent it. |
| 4 | Being bullied at school / cold treatments | 小学校のころクラスメイトにいじめられた。毎日のように死ね、消えろなど言われ、クラス内で私がいじめられていることについて話し合いが行われ、話し合いの中で私がすべて悪いと言われ、教師も何も解決のために動いてくれなかった。 | I was bullied by my classmates in elementary school. They told me things like “die” and “disappear” almost every day. There was even a discussion in class about the bullying, but during it, I was blamed for everything, and the teacher did nothing to resolve the situation. |
| 5 | Troubles with relatives or friends | 親戚のおばさん。相続で揉めてるのですが、祖母の年忌に家まで来て大声でわーわーいってきて、隣人もどうしたのか心配して家に来るくらいでした。ほんとに許せない | My aunt. We’ve been having disputes over an inheritance, and she even came to our house during my grandmother’s memorial, shouting loudly. It was so bad that the neighbors came over, worried about what was happening. I truly cannot forgive her. |
| 6 | Violence / backbiting | 中学生のとき、全校集会で一部の生徒が騒いでいたところ、教師から冤罪で私が犯人にされ前に出されて皆の前で腹を殴られた | When I was in junior high school, some students were causing a disturbance during a school assembly, but I was falsely accused by a teacher, brought to the front, and punched in the stomach in front of everyone. |
