# Supplementary material for: The feeling of “Urami”: A structural topic modeling approach
Source: PLoS One. 2026 May 26;21(5):e0349193. doi: 10.1371/journal.pone.0349193 (PMC13210193; doi:10.1371/journal.pone.0349193)
Supplement: S4 Table — Bold values indicate that the effect of the prevalence covariate was statistically significant at the 5% level. (DOCX) [file pone.0349193.s009.docx]

**S4 Table. Estimated Values (b), Standard Errors (SE), and p-Values for prevalence covariates in Each topic regarding situation of *urami*.** Bold values indicate that the effect of the prevalence covariate was statistically significant at the 5% level.

| Scale (prevalence covariate) | | Topic | *b* | *SE* | *t* | *p* | *Corrected p* |
| --- | --- | --- | --- | --- | --- | --- | --- |
|  | Age | 1 | 0.00 | 0.00 | 0.25 | .800 | .900 |
|  |  | 2 | 0.00 | 0.00 | 2.31 | .021 | .081 |
|  |  | **3** | **0.00** | **0.00** | **3.46** | **.001** | **.009** |
|  |  | **4** | **0.00** | **0.00** | **-3.44** | **.001** | **.009** |
|  |  | 5 | 0.00 | 0.00 | -2.14 | .033 | .105 |
|  |  | 6 | 0.00 | 0.00 | -1.39 | .166 | .355 |
|  | Five Facet Mindfulness Questionnaire | 1 | 0.00 | 0.01 | 0.15 | .882 | .953 |
|  | Describing | 2 | -0.01 | 0.02 | -0.69 | .489 | .714 |
|  |  | **3** | **0.04** | **0.01** | 3.06 | **.002** | **.015** |
|  |  | 4 | 0.00 | 0.01 | -0.17 | .866 | .953 |
|  |  | 5 | 0.00 | 0.01 | 0.31 | .756 | .887 |
|  |  | 6 | -0.03 | 0.01 | -2.55 | .011 | .059 |
|  | Interoceptive Accuracy Scale | 1 | 0.04 | 0.02 | 2.5 | .013 | .064 |
|  |  | 2 | -0.03 | 0.02 | -1.41 | .159 | .355 |
|  |  | 3 | 0.04 | 0.02 | 2.07 | .039 | .117 |
|  |  | 4 | 0.01 | 0.02 | 0.63 | .532 | .734 |
|  |  | 5 | -0.02 | 0.02 | -1.37 | .171 | .355 |
|  |  | 6 | -0.03 | 0.02 | -1.71 | .088 | .238 |
|  | Interoceptive Attention Scale | 1 | 0.00 | 0.01 | 0.11 | .909 | .962 |
|  |  | 2 | -0.03 | 0.02 | -1.40 | .162 | .355 |
|  |  | 3 | -0.03 | 0.01 | -2.22 | .027 | .097 |
|  |  | 4 | -0.01 | 0.01 | -0.58 | .565 | .744 |
|  |  | 5 | 0.02 | 0.02 | 1.06 | .290 | .475 |
|  |  | **6** | **0.05** | **0.01** | **3.32** | **.001** | **.009** |
|  | Hypervigilant-oblivious narcissism scale | 1 | 0.01 | 0.01 | 0.93 | .354 | .535 |
|  | Oblivious narcissism | 2 | -0.01 | 0.02 | -0.48 | .633 | .795 |
|  |  | 3 | 0.02 | 0.01 | 1.11 | .269 | .454 |
|  |  | 4 | 0.01 | 0.01 | 0.36 | .719 | .863 |
|  |  | 5 | -0.01 | 0.01 | -0.50 | .619 | .795 |
|  |  | 6 | -0.02 | 0.01 | -1.15 | .252 | .454 |
|  | Hypervigilant narcissism | 1 | 0.00 | 0.01 | -0.4 | .688 | .844 |
|  |  | 2 | 0.00 | 0.01 | 0.04 | .965 | .967 |
|  |  | **3** | **-0.05** | **0.01** | **-3.97** | **< .001** | **< .001** |
|  |  | 4 | 0.01 | 0.01 | 0.61 | .544 | .734 |
|  |  | 5 | 0.00 | 0.01 | -0.29 | .775 | .890 |
|  |  | **6** | **0.05** | **0.01** | **4.21** | **< .001** | **< .001** |
|  | Transgression-Related interpersonal Motivations Inventory | 1 | 0.01 | 0.01 | 0.92 | .357 | .535 |
|  | Revenge | 2 | -0.02 | 0.01 | -1.12 | .263 | .454 |
|  |  | **3** | **-0.04** | **0.01** | **-3.43** | **.001** | **.009** |
|  |  | 4 | 0.01 | 0.01 | 0.64 | .521 | .734 |
|  |  | 5 | 0.02 | 0.01 | 1.27 | .205 | .410 |
|  |  | 6 | 0.02 | 0.01 | 2.15 | .032 | .105 |
|  | Forgiveness of Others Scale | 1 | 0.00 | 0.02 | 0.09 | .930 | .966 |
|  | Forgiveness | 2 | 0.00 | 0.03 | 0.04 | .967 | .967 |
|  |  | 3 | 0.05 | 0.02 | 2.35 | .019 | .079 |
|  |  | 4 | 0.03 | 0.02 | 1.22 | .224 | .423 |
|  |  | 5 | -0.03 | 0.02 | -1.21 | .227 | .423 |
|  |  | 6 | -0.05 | 0.02 | -2.40 | .017 | .077 |
|  | Scale Measuring a Sense of Generalized Self-Efficacy | 1 | 0.02 | 0.01 | 1.92 | .055 | .156 |
|  |  | 2 | -0.01 | 0.01 | -1.00 | .318 | .505 |
|  |  | 3 | 0.03 | 0.01 | 2.54 | .011 | .059 |
|  |  | 4 | 0.02 | 0.01 | 1.46 | .146 | .355 |
|  |  | 5 | -0.02 | 0.01 | -1.45 | .147 | .355 |
|  |  | **6** | **-0.03** | **0.01** | **-2.77** | **.006** | **.040** |
