# Supplementary material for: The feeling of “Urami”: A structural topic modeling approach
Source: PLoS One. 2026 May 26;21(5):e0349193. doi: 10.1371/journal.pone.0349193 (PMC13210193; doi:10.1371/journal.pone.0349193)
Supplement: S5 Table — Excerpts were selected based on their high interpretability and ease of translation into other languages. (DOCX) [file pone.0349193.s010.docx]

| Topic | Topic name | Original documents | Documents translated in English |
| --- | --- | --- | --- |
| 1 | Injustice / unreasonable | 理不尽な目に合わされた時に沸き起こる感情だと思う。納得できない、自分ばかりが損をしたと感じた時に捕らわれる感情。 | My partner suddenly broke up with me because of their infidelity. Soon after, they got married and even sent me an invitation to their wedding. |
| 2 | Desiring assailant’s unhappy / intention to revenge | 私に対して酷い行いをした相手に対して復讐したいと思ったり、嫌いな相手の不幸を願うことや、天罰が当たれば良いと思うことです。 | I think it is a feeling that arises when one is treated unfairly. It is the emotion that takes hold when you cannot accept the situation and feel that you alone have been treated unfairly or suffered a loss. |
| 3 | Arising feeling | 心の底からわきおこるような、感情。ふだん、なかなか出てこない心の奥底にしまいこんでいる気持ち。 腹が立った時に出る感情。色で表すと真っ赤な感じ。 言葉は悪いかもしれないが、キレるという言葉がふさわしいかもしれない。 | A feeling that rises from the depths of one’s heart—something usually buried deep inside and rarely comes out. It surfaces when you become angry. If expressed as a color, it would be bright red. The word “losing one’s temper” might describe it well. |
| 4 | Being irritated | 自分の思い通りにならなかったり、相手が期待したとおりの行動を取らなかったときに感じる憤りや苛立ちのこと。 不安や心配、悲しみなどから生じる負の感情で、心の奥底でメラメラと燃えたぎるようなネガティブな熱い感情 | A feeling of anger or frustration that arises when things do not go as you want or when others do not act as expected. It is a negative emotion stemming from anxiety, worry, or sadness, and burns deep inside like an intense, simmering heat. |
| 5 | Persistence / unforgiveness | 許す事が出来ない、一生忘れられない出来事が脳裏に刻まれていて、とても自分が傷付いているし復讐をしたくなる思い。 憎しみ 頭から消えない | An unforgivable, unforgettable event is etched in your mind, leaving you deeply hurt and filled with a desire for revenge. It is a feeling of hatred that you cannot get out of your head. |
| 6 | Sense of being harmed by betrayal or a violation of one’s dignity | 私自身との付き合いにおいて、表面上では常にいい顔をして非常に親密な態度をとっておきながら、裏に回ると表の顔とは真逆に悪口を言いふらしている友人の現実を知った時の感情。 | The feeling you experience when you discover that a friend who always acted kind and very close to you in public was, behind your back, spreading insults and speaking badly about you. |
| 7 | Intense negative emotions | 心からの怒り、何かに当たりたい怒り、信じていた者に裏切られたという怒りが一辺に襲いかかってくる気持ち。 | A feeling in which deep anger, the urge to lash out at something, and the anger of being betrayed by someone you trusted all come rushing in at once. |
| 8 | Helpfulness / having nowhere to direct one’s emotions | 自分の大切な人が傷つけられたり、大事なものが壊されたりなど、理不尽な形で咎められた時に起こる気持ち やれなければいけないことができなかったり、何度も同じ過ちを繰り返す自分に対して起こる感情 | A feeling that arises when someone important to you is hurt, something you value is damaged, or you are blamed unfairly. It can also arise toward yourself when you fail to do what you should or keep repeating the same mistakes. |
| 9 | Disgusting / disappointment | 自分以外の人に対して、嫌い、いやなことをされて接するのがいや、顔さえも見たくないというような否定的な気持ち。 いやな出来事が起きた後も、ずっと気持ちをひきずってしまうような感情。 いやな出来事以外にもう何も起きてほしくないような気持ち | A negative feeling toward others—disliking them, feeling upset by what they have done, not wanting to interact with them, and not even wanting to see their face. It is also an emotion that lingers long after an unpleasant experience, leaving you wishing that nothing else bad would happen. |
| 10 | Irritation / arousal / bodily sensations | 頭に血が上り、心臓の鼓動も激しくなり、大きな声で言葉を発したくなる衝動にかられること こんな力がでるんだという悪い意味でのパワーがみなぎる感じ 精神が興奮する事。すぐに収めるのが難しい感情。その時に発する言葉や、態度は後悔することが多い。 | A state in which your blood rushes to your head, your heart starts pounding, and you feel an urge to shout. It feels like a surge of intense, negative energy, as if a powerful force is rising within you. Your mind becomes highly agitated, and it is difficult to calm down quickly. The words you say and the actions you take in that moment are often things you later regret. |
| 11 | Suddenness / uncontrollable | 人物や物事に対して、激しく憎たらしい感情を抱くこと。破壊したくなるような、衝動がある。強い興奮を伴う。 | A feeling of intense hatred toward a person or situation, accompanied by an impulse to destroy and a strong sense of agitation. |
