# Supplementary material for: The feeling of “Urami”: A structural topic modeling approach
Source: PLoS One. 2026 May 26;21(5):e0349193. doi: 10.1371/journal.pone.0349193 (PMC13210193; doi:10.1371/journal.pone.0349193)
Supplement: S6 Table — Excerpts were selected based on their high interpretability and ease of translation into other languages. (DOCX) [file pone.0349193.s011.docx]

| Topic | Topic name | Original documents | Documents translated in English |
| --- | --- | --- | --- |
| 1 | Troubles in medical scene / troubles with relatives | 母の担当医に無視され（男の人には説明や会話があるが女性に対してはまったく話さない）、症状から脳疑を疑って担当医に訴えてもまったく無視され続け、別の病院での診断は脳梗塞と分かった後のその医者の反応は「あ、そう」だったとき | The feeling you have when your mother’s doctor ignores you—speaking and explaining things to men but not to women at all—and continues to dismiss your concerns even when you suspect a brain condition from her symptoms, only for another hospital to later diagnose it as a stroke, and the doctor’s reaction is simply, “Oh, I see.” |
| 2 | Getting bullied | 学生時代、同じクラスの人間からいじめを受けた。陰口やクラス内に飾ってあった私の展示物に対し傷を付けるなど様々な嫌がらせをされた。 | During my school days, I was bullied by classmates. They spread rumors behind my back and even damaged my work that was displayed in the classroom, along with various other forms of harassment. |
| 3 | Sighting of inappropriate behaviors | 公園で酒を飲み酔っ払って悪ふざけした若者集団がゴミを放置し、ビンを投げて割れるのを喜び、木々を傷つけていたこと | A group of young people, drunk in a park, behaved recklessly—leaving trash behind, throwing bottles and enjoying watching them shatter, and damaging the trees. |
| 4 | Troubles in transportation | 車で4車線の道を直進して運転している時に左レーンの車が急に車線変更してき、あわやぶつかりそうになった時 | While I was driving straight on a four-lane road, a car in the left lane suddenly changed lanes and nearly caused a collision. |
| 5 | Cold treatments | 義父のお葬式に行ったときに私だけ身内の扱いをしてもらえず、遠い親戚のような扱いを受けた。またその後の参りでも義母や義兄から常に夫と席を離されて夫婦の扱いをしてもらえなかった。 | When I attended my father-in-law’s funeral, I was not treated as a family member and was instead treated like a distant relative. Even afterward, during memorial visits, my mother-in-law and brother-in-law kept separating me from my husband, and I was never treated as part of the couple. |
| 6 | Distrustfulness to others | 妊娠して重度のつわりでほとんど食べる事ができず寝たきり状態の私を置いて遊びの予定を変更できないからと夫が休日に遠出した | While I was pregnant and suffering from severe morning sickness—unable to eat and mostly bedridden—my husband went out on a trip on his day off, saying he couldn’t change his plans. |
| 7 | Violence / backbiting | 自分以外の人が原因で起こったトラブルがあり、それで怒った相手から、何の理由も言われず、いきなり当たってこられたときに激しく動揺し、関係者もすぐにはその原因を教えてくれなかった | There was a problem caused by someone else, and then the person who got angry suddenly took it out on me without any explanation. I was deeply shaken, and even the people involved would not immediately tell me the reason. |
| 8 | Power harassments / denial of one’s personality | 職場で不利益が生じるように足を引っ張られたり、あからさまなパワーハラスメントを受けたりしたこと。本人は一時の感情での行動かもしれないが、マイナスを被った側としては許すことができない。 | Being undermined at work in ways that cause disadvantage, or being subjected to obvious power harassment. The person responsible may have acted on temporary emotions, but for the one who suffers the harm, it is something that cannot be forgiven. |
| 9 | Troubles with relatives / harm to family | 父が亡くなったときに財産をほとんど相続した兄が、相続したとたん急に態度が変わり母に冷たくなったこと | When my father passed away, my older brother inherited most of the assets, and as soon as he did, his attitude suddenly changed and he became cold toward our mother. |
| 10 | Power harassments | 新しい職場に移った時、上司から言葉によるパワハラを受けた。はじめは耐えていたが限界を超え、うつ病になった。それにより退職を余儀なくされ、社会復帰が難しくなった。 | When I moved to a new workplace, I was subjected to verbal harassment by my supervisor. At first, I endured it, but eventually it exceeded my limits and I developed depression. As a result, I was forced to resign, and returning to society became difficult. |
| 11 | Troubles with money | 知り合いにお金を貸した後で、音信不通になってしまいお金が返って来なかった時に恨むような気持がありました。 | After lending money to an acquaintance, they suddenly cut off contact and never repaid it, which left me feeling resentful. |
